# Flexible active electrode arrays with ASICs that fit inside the rat’s spinal canal

**DOI:** 10.1007/s10544-015-0011-5

**Published:** 2015-10-14

**Authors:** Vasiliki Giagka, Andreas Demosthenous, Nick Donaldson

**Affiliations:** Department of Medical Physics and Biomedical Engineering, University College London, London, WC1E 6BT UK; Department of Microelectronics, Delft University of Technology, Delft, 2628 CD The Netherlands; Department of Electronic and Electrical Engineering, University College London, London, WC1E 7JE UK

**Keywords:** Active flexible electrode arrays, Application specific integrated circuits, Epidural stimulation, Laser patterned microelectrodes, Neurorehabilitation, Rivet bonding

## Abstract

Epidural spinal cord electrical stimulation (ESCS) has been used as a means to facilitate locomotor recovery in spinal cord injured humans. Electrode arrays, instead of conventional pairs of electrodes, are necessary to investigate the effect of ESCS at different sites. These usually require a large number of implanted wires, which could lead to infections. This paper presents the design, fabrication and evaluation of a novel flexible active array for ESCS in rats. Three small (1.7 mm^2^) and thin (100 μm) application specific integrated circuits (ASICs) are embedded in the polydimethylsiloxane-based implant. This arrangement limits the number of communication tracks to three, while ensuring maximum testing versatility by providing independent access to all 12 electrodes in any configuration. Laser-patterned platinum-iridium foil forms the implant’s conductive tracks and electrodes. Double rivet bonds were employed for the dice microassembly. The active electrode array can deliver current pulses (up to 1 mA, 100 pulses per second) and supports interleaved stimulation with independent control of the stimulus parameters for each pulse. The stimulation timing and pulse duration are very versatile. The array was electrically characterized through impedance spectroscopy and voltage transient recordings. A prototype was tested for long term mechanical reliability when subjected to continuous bending. The results revealed no track or bond failure. To the best of the authors’ knowledge, this is the first time that flexible active electrode arrays with embedded electronics suitable for implantation inside the rat’s spinal canal have been proposed, developed and tested *in vitro*.

## Introduction

### Background

Recently epidural spinal cord electrical stimulation (ESCS) has been shown to restore supra-sacral control of muscles paralysed by spinal cord injury (SCI). Harkema et al. (2011) reported that some hitherto paralysed muscles in patients, who had suffered traumatic injuries, can be activated normally while others can be contracted by volition only while stimulation is being applied. This was an exciting discovery but one that raised many questions for neuroscience and rehabilitation. It is important to examine how this latent neuroplasticity can be used to benefit the large number of people who have suffered SCI.

van den Brand et al. (2012) conducted methodical experiments on the effect of epidural stimulation applied after causing spinal injuries in rats. The electrodes were connected by head connectors to an external stimulator during the therapy. Stimulation was sometimes combined with application of drugs. In their paper they showed that all the rats in the experiment recovered the ability to move their lower limbs in order to reach a reward. During the training and tests, the fore-limbs were held above the ground and a set proportion of their body weight was supported from a robot which itself moved to avoid applying any horizontal force to the animal so that all movement along the walkway was due to the animal. The animals could not only walk but also ascend steps and step-over hurdles, clearly demonstrating supra-sacral control. However, these rats were trained for only 30 min per day, a human therapist being required as well as the robot system. These experiments should be extended to investigate the advantages of increasing the number of stimulation sites, and to apply the stimulation for long periods, such as while the experimental rats are living in a cage with other animals, to see whether recovery can be greater or faster. To enable such experiments, electrode arrays, as opposed to the conventional pairs of electrodes (Abdo et al. 2011; Zhou et al. 2012), can be used to investigate the effect of ESCS at different sites. These arrays must be driven by an implanted stimulator that will run autonomously while the rat is free to move in the cage.

The development of epidural electrode arrays for chronic testing in rats presents special challenges compared to other parts of the body. The main issues relate to the limited cross-sectional area at the implantation site [3 mm wide, 300 μm thick (Hodgetts et al. 2009)], the fixation of the implant, and the vulnerable nature of the spinal cord. Movements of the spinal cord with respect to the dura and the vertebrae, as well as movements of the vertebrae with respect to each other pose a major challenge for the implant design and its mechanical properties (Prochazka and Mushahwar 2007). Furthermore, there is the risk to damage the delicate spinal cord due to compression from the implanted materials.

Passive (without embedded electronics) electrode arrays for ESCS in rats have been recently reported (Giagka et al. 2012; Minev et al. 2015). Passive arrays, although useful for initial testing have two main disadvantages. The first is that when a large number of electrodes needs to be independently controlled they quickly become very complex to fabricate, unreliable (Giagka et al. 2013) or even unsuitable for long-term implantation (Thelin et al. 2011) due to the large number of connections required. They might fail, imperil perfusion, or result in infections or tissue damage. The second disadvantage of passive implants is that they usually must be connected to external electronics, hence their use is limited to testing protocols that involve animals tethered to the testing setup.

The small size of application specific integrated circuits (ASICs) can be exploited by assembling the custom designed silicon microchips on the passive electrode arrays (i.e., active arrays). Depending on the ASIC’s functionality, the active microimplants could potentially lead to devices free from external equipment, suitable for testing in freely moving animals.

This paper presents the design, fabrication and characterization of active flexible epidural electrode arrays for ESCS in rats. The implants are made of polydimethylsiloxane (PDMS) substrates and feature 12 platinum-iridium (PtIr) electrodes which can be independently selected in any possible configuration. Embedded ASICs minimize the required power supply and control signal connections to only three, while featuring rapid parameter adjustment and ultralow power consumption. To the best of the authors’ knowledge, this is the first time that active electrode arrays with microelectronics suitable for implantation inside the rat’s spinal canal have been developed and tested. The rest of this paper is organized as follows. The following subsection discusses the specifications posed by the implantation site and presents the proposed implant design. Section 2 describes the selected materials, the operation of the electrode driving ASICs, the pre-fabrication ASIC processing (thinning and laser-shaping), and the process of electrical rivet bonding for assembling thin ASICs. It details the entire proposed fabrication flow, the control of the devices by the user, and the related methods for mechanical and electrical evaluation of the fabricated prototypes. Results are reported in Section 3. Discussion and conclusions, in Sections 4 and 5, complete the paper.

### Implantation-site related specifications and proposed design

The concept of integrating electronic circuits close to their respective electrodes is not new and active electrode arrays have been previously reported for a variety of applications. Lithographically patterned probes and arrays on silicon substrates, where custom circuits for processing are included were reported (Wise et al. 2008 and Maynard et al. 1997). These can be batch-fabricated and have been successfully used for acute and chronic studies. However, their stiffness makes them unsuitable for chronic implantation inside the spinal canal. Electrode arrays on flexible substrates (polyimide, parylene or PDMS) have also been investigated, and some hybrid active implants have been reported (Stieglitz et al. 2005; Ramachandran et al. 2006; Guo et al. 2013). Recently, a parylene-based epidural electrode array for rats was reported (Gad et al. 2013). This features 27 PtIr electrodes, which are driven in bipolar configurations by an external controller connected via 12 wires to 10 implanted multiplexing chips. These are implemented by discrete off-the-shelf components on a 10.3 × 33.2 mm printed circuit board (PCB). This large PCB is only suitable for mounting outside the spinal canal, on top of the vertebrae.

**Fig. 1:**
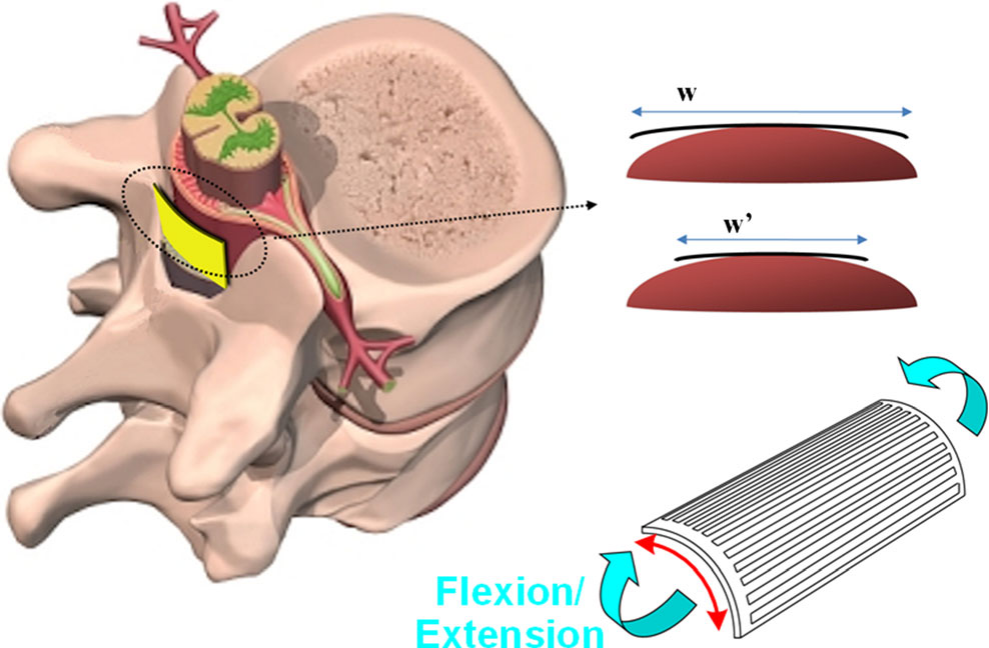
Epidural array with longitudinal metal tracks, here shown subjected to bending when the rat extends its spine **(** ***bottom right*** **)**. w and w’ highlight how narrower implants conform better to the natural curvature of the spinal cord

A device suitable for implantation inside the rat’s epidural space should be less than 300 μm thick (Hodgetts et al. 2009). This limitation, while not a major challenge for passive devices, disqualifies standard electronic drivers from being close to their respective electrodes; they instead need to be connected through several long tracks running along the implant, as in Gad et al. (2013). For an array with *N* independent electrodes, *N* long tracks are necessary for their control. This soon poses a limitation on the number of independently accessible sites, depending on the resolution of the fabrication process. Moreover, the larger this number, the wider the implant becomes and the likelihood of failure increases dramatically. The diameter of the rat’s spinal cord is about 3 mm (Hodgetts et al. 2009) and previous attempts using implants that covered most of this surface showed that the deformation caused by the movements of the rat on the wide implant resulted in track failure (Giagka et al. 2012). The plane array, after being bent around the spinal cord, as indicated by the curved array in the bottom right part of Fig. 1, along the red arrow, could not then also be bent without high mechanical stress when the rat flexed or extended its spine towards the direction of the blue arrows in Fig. 1 (Giagka et al. 2013). For PDMS-based arrays the main component limiting the flexibility of the device are the metal tracks. The use of thin metal layers (in the nanometer scale), could produce implants of increased flexibility. However, interconnecting these films reliably to the more rigid wires required for communication is currently a major unsolved challenge. Alternatively, this situation could be avoided by designing narrower implants. This expectation has been supported by comparative mechanical lab tests using similar wide and narrow polyimide-based arrays (Detemple 2012).

**Fig. 2:**
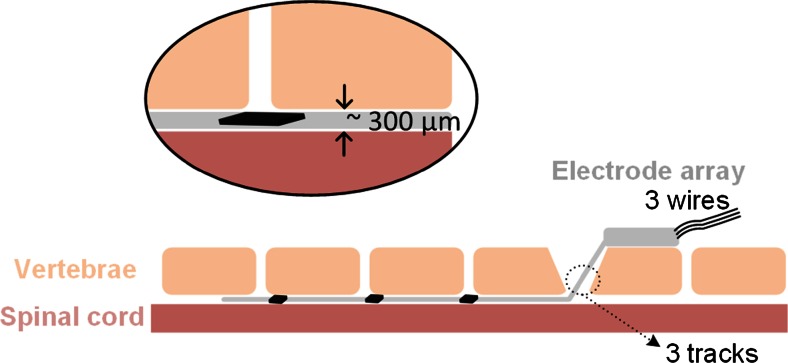
Layout of the implant in respect to the implantation site. The electrode array is represented by the *grey* structure entering the spinal canal through the laminectomy. The electrodes are located in the implant’s main body, in contact with the spinal cord. Close to them, *black* structures represent the small driver ASICs

**Fig. 3:**
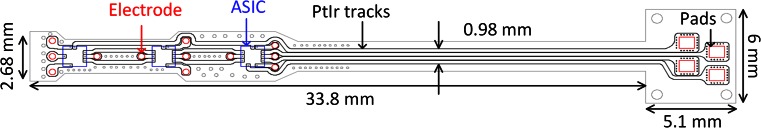
Design details of the active arrays with ASICs along the spinal cord. *Black lines* indicate the metal and *grey lines* the overall shape of the array. The *blue rectangles* correspond to the ASICs (1.324 × 1.324 mm each) and *red lines* indicate the openings on the PDMS for electrodes (0.4 mm^2^ each) and pads (850 × 710 μm each)

To overcome these problems, this work proposes drivers that are adjacent to their electrodes, thus reducing not only the number of communication wires, but also the number of long tracks, as shown in Fig. 2. To implement this idea however, thin, rather than standard thickness, ASICs are necessary. These ASICs represent rigid islands on the array hence a minimal area is desirable. Figure 3 illustrates the design details of the proposed arrays, respecting the above specifications, where independent access to 12 electrodes (running all the way from *L*2 down to *S*1 spinal segments, associated with the control of the hind limb (Watson and Sidhu 2009) is achieved by only three long tracks. The fourth track visible on the figure can provisionally be connected to an extra electrode implanted subcutaneously far from the array which may be used when monopolar stimulation is required. In this design the width of the stiff part of the ribbon of tracks exiting the spinal canal is kept very narrow – approximately 1 mm – while the width of each of the individual tracks does not need to be less than 200 μm, which ensures that they remain strong for chronic use. In contrast, a similar passive device with the same track width would be about 3.15 mm wide, already exceeding the 3 mm available space. Previous work done at the Courtine-Lab at École Polytechnique Fédérale de Lausanne (EPFL), and in Giagka et al. (2012, 2013) helped with the definition of the system specifications related to the number of electrodes (to allow multisite stimulation) and their size (suitable to safely deliver the desired currents), as well as the electrode configuration.

## Materials and methods

### PDMS and PtIr foil

PDMS has been extensively used in the fabrication of implantable electrodes. It can be laser-patterned (Schuettler et al. 2005), is softer and more flexible compared to polyimide or parylene, and it has also been shown that it can be suitable for long term encapsulation of silicon ASICs (Vanhoestenberghe and Donaldson 2013). It was selected for this application to provide an insulating substrate that would ideally adjust to the shape of the spinal cord and move in sympathy with it. A two-part, low viscosity translucent PDMS was preferred (MED4-4220, NuSil silicone technology, Carpinteria, CA, USA).

In ESCS the charge injection required to elicit neuron response is large due to the thick dura layer. PtIr exhibits a large charge storage capacity (CSC) (alloys of platinum (Pt) with 10–30 % iridium (Ir) have similar CSC to pure Pt (Robblee et al. 1983), in the range of 50–350 μC/cm^2^ (Merrill et al. 2005). Assuming the minimum reported CSC of 50 μC/cm^2^, the 0.4 mm^2^ electrode area would suffice to safely deliver the maximum charge of 200 μs × 1 mA = 0.2 μC. Hard PtIr foil (Pt80/Ir20, 12.5 μm thick, Goodfellow Cambridge Ltd, Huntington, UK) was selected to form the conductive parts of the implant. It was preferred to previously reported pure Pt (Giagka et al. 2012, 2013; Schuettler et al. 2005) due to its better fatigue resistance. PtIr has been previously used for implants in other forms and is known to be well tolerated by the tissue (Merrill et al. 2005).

### Electrode driving ASICs

The three electrode driving ASICs that are embedded on the array have been designed to feature a very small layout area and minimal power consumption. They receive all the information required for their operation through three inputs, namely V_in_, V_SS_ and I_in_.

In an alternative implementation where only one larger ASIC would control all electrodes, the ASIC, due to area restrictions, would have to be placed at the end of the array which lies outside the spinal canal (this arrangement and the layout of such an ASIC have also been discussed in Giagka et al. 2014c). This arrangement would still reduce the number of communication wires, but the number of long tracks running along the length of the array to the electrodes would remain high (12). Hence, in this work three smaller ASICs were employed instead, which were designed to be located next to their respective electrodes.

**Fig. 4:**
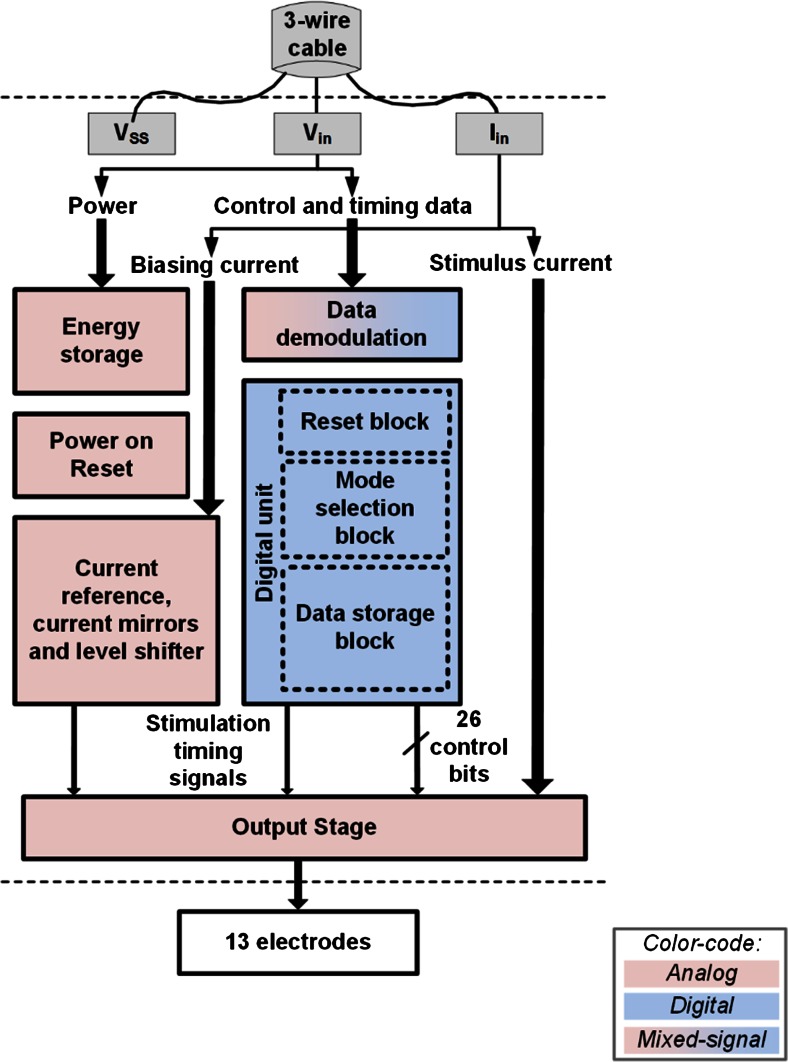
Block level diagram showing the different components of the ASIC. The ASICs can independently control the 12 electrodes on the array and the extra subcutaneous electrode for monopolar configurations; the *color-code* indicates the main nature of the design for each block

To keep the number of inputs small, power and data are multiplexed and sent through the first two lines (data are sent as pulse width modulated voltage pulses over the power line). This is enabled by an on-chip storage capacitor. The third line delivers the stimulus current, up to 1 mA, with one or two pulses to give monophasic or biphasic electrode currents respectively. This is externally generated to keep the silicon area small, and most importantly, to reduce the power consumption (and hence heat dissipation) close to the spinal cord. The on-chip analog circuits have been designed for minimum power dissipation. An asynchronously controlled digital unit provides maximum versatility on the control of the stimulation timing, while its design also ensures minimum unwanted power consumption by avoiding on-chip clocks. As a result a maximum of only 114 μW is consumed by the whole 3-ASIC-system during a full biphasic stimulus cycle. The ASICs have an output voltage compliance of up to 25 V, to accommodate for high impedance loads or high currents. A block level diagram showing the components of the circuit is presented in Fig. 4. Further details about the electronic design and performance of the ASICs can be found in Giagka et al. (2014c).

All three ASICs have different layouts and work together as one system able to drive 13 electrodes (12 on the array and one extra subcutaneous electrode) independently, in any possible configuration. Interleaved stimulation is also supported, allowing more pulses of different characteristics, all delivered from a single current source, at repetition rates of up to 100 pulses per second (pps). Each ASIC can be used independently of the others if needed (e.g., for a smaller system). All ASICs receive and store the same information from the external control unit, however each ASIC only services specific electrodes (defined by a unique electrode ID). Their footprint is only 1.75 mm^2^ with a square layout. They include large (150 × 150 μm, with 100 μm spacing) custom designed pads to facilitate manual microassembly on the flexible array. The prototype chips were fabricated using XFAB XP018, a 0.18 μm high-voltage CMOS technology.

### ASIC thinning and laser shaping

#### Individual die thinning

The dice received from the foundry were approximately 1 mm thick. To satisfy the restrictions for the total implant volume, a dice thinning target of 100 μm was set. ASICs of this thickness are already thin enough for this application, and are, at the same time, strong enough to handle.

The thinning of silicon chips is usually done at wafer level, and processes such as mechanical grinding followed by a chemical stress-relief step are commonly employed (Burghartz et al. 2010; Hawkins et al. 1987). The purpose of this final step is to reduce the backside damage caused by the previous coarse step, which could compromise the die strength (Kröninger and Mariani 2006). Such procedures are today well established and reported thicknesses are as little as 25 μm (Burghartz et al. 2010). However, in cases where ASICs are parts of multi-project wafers (as in this implementation) the thinning needs to be done at individual die level where the handling and processing requirements differ significantly. For this application, a novel method has been developed that is suitable for controlled thinning of individual small dice (1 mm^2^). The method is based exclusively on mechanical polishing. This ensures that no special care needs to be taken to avoid damaging the active die area due to chemicals – this could be especially complicated for such small areas.

**Fig. 5:**
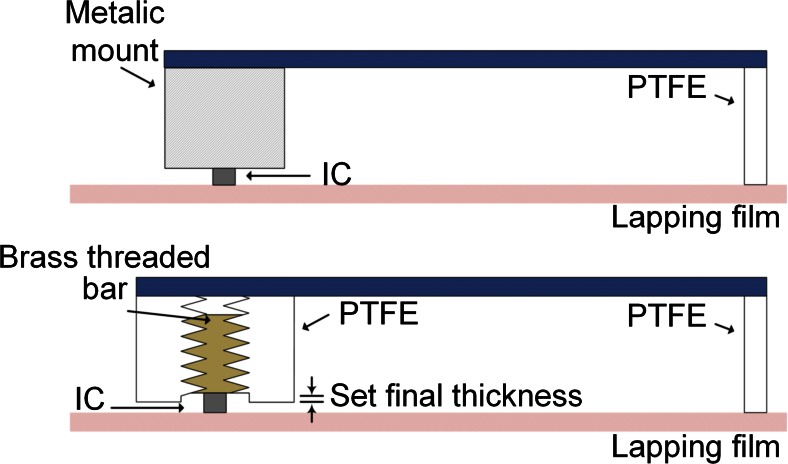
Thinning kinematics for the commercial **(** ***top*** **)** and custom-made **(** ***bottom*** **)** setup; the new die holder is made of polytetrafluoroethylene (PTFE). Due to the fact that PTFE is polished at much slower rates compared to silicon, the holder’s bottom layer serves as a stop layer for the thinning. The PTFE rod shown, together with another one (not shown here), ensure the alignment of the die surface against the polishing film

The method changes the standard kinematics of the thinning that apply when commercial equipment is used (as in Fig. 5, top). A new, custom designed addition to the commercial setup serves as the die holder while only removing silicon that protrudes the created cavity (as in Fig. 5, bottom). In this manner, the user is able to accurately set the final thickness of the dice by adjusting the z-axis position of a threaded bar (on which the die is attached) under a microscope. Characterization tests on eight samples – for which the target thickness was set to 80 μm – showed that a mean thickness of 79.8 μm, with a standard deviation of 9.2 μm, was achieved. In addition, by switching to smaller grit size when lapping in the last processing step, very smooth surfaces can be achieved (surface roughness values of 1.75 nm), thus improving the mechanical strength of the dice without the need of chemical processing. More details and further results related to this method were presented in Giagka et al. (2014a).

#### Laser shaping for smoother layout

Typically, dice are separated from a wafer by means of mechanical sawing, which produces rectangular chips. However, the sharp corners that are present after such processing are likely to tear the flexible PDMS surrounding them and lead to implant failure. To avoid this a smoother profile is desirable, which can be achieved by post-processing the separated die with laser-cutting. This method was used after thinning to modify the final die layout and avoid stress concentrations in the PDMS at the corners. The sharp rectangular corners were smoothed and shaped to a 0.17 mm radius.

### Electrical rivet bonding on thin ASICs

Several approaches have been proposed for the assembly of thin electronics on flexible substrates. These include flip chip bonding by means of reflow soldering, thermocompression processes (Banda et al. 2008; Zhang et al. 2009) and polymeric adhesives (Chen et al. 2000), gold electroplating (Govaerts et al. 2009), as well as conductive pastes (Marinov et al. 2012).

The microflex assembly technique (Stieglitz et al. 2000), also referred to as electrical rivet bonding, has been previously used to connect standard thickness electronics in hybrid systems (Meyer et al. 2001). In rivet bonding, conductive tracks are thermosonically bonded on a substrate using gold ball studs as microrivets through via holes on the tracks. The bond created connects the two pieces together both electrically and mechanically. All the materials implemented here have been previously used for implants, and no additional equipment is necessary other than a wire bonder (also an automated ball/stud bumper could be used), which makes this process very appealing. The reported electrical and mechanical properties of the bonds are satisfactory and the process is stable and with a high yield (Stieglitz et al. 2000).

Extending the existing studies, this work shows that the method is also suitable for bonding on thin ASICs, provided the chips are properly supported throughout the process. In addition the mechanical strength of the bonds created on standard aluminum (Al) pads was evaluated and a parameter optimization study was conducted (Giagka et al. 2014b).

This paper is the first to show the use of this process for assembling thin dice on a flexible PDMS implant. Two vertically aligned gold ball studs (each approximately 90 μm in diameter) were employed for each bond, one above and one below the 150 × 150 μm PtIr pad (it is estimated that a misalignment of approximately up to 25 μm between the PtIr and ASIC pads, and/or the positioning of the first gold stud could be tolerated.) The parameters used for the rivet bonding are those in Table 1.

**Table 1:** Parameters for electrical rivet bonding

Parameter	Value
Ball size (bsu^a^)	4.2
Hole size (μm)	75 μm
Gold thread diameter (μm)	30 μm
Temperature (°C)	120 °C
Power (bsu^a^)	4.5
Time (bsu^a^)	3
Force (bsu^a^)	4.5

^a^ *bsu* bonder specified units

### Proposed fabrication method

The proposed fabrication method of the active array comprises three main processes: (I) fabrication of the electrode interconnects, (II) microassembly of the ASICs, and (III) final encapsulation. Each process comprises several different steps. The flow of the basic processes is summarized below:

- *Fabrication of the electrode interconnects (top-layer-first approach):*
  - Formation and shaping of the top PDMS layer.
  - Lamination of metal and shaping of the conductive lines, pads and electrodes.
  - Formation and shaping of the bottom PDMS layer.
- *Microassembly of the ASICs:*
  - Bumping of the thin dice.
  - ASICs and flexible array alignment and assembly.
- *Final encapsulation:*
  - Bottom layer.
  - Top layer.

**Fig. 6:**
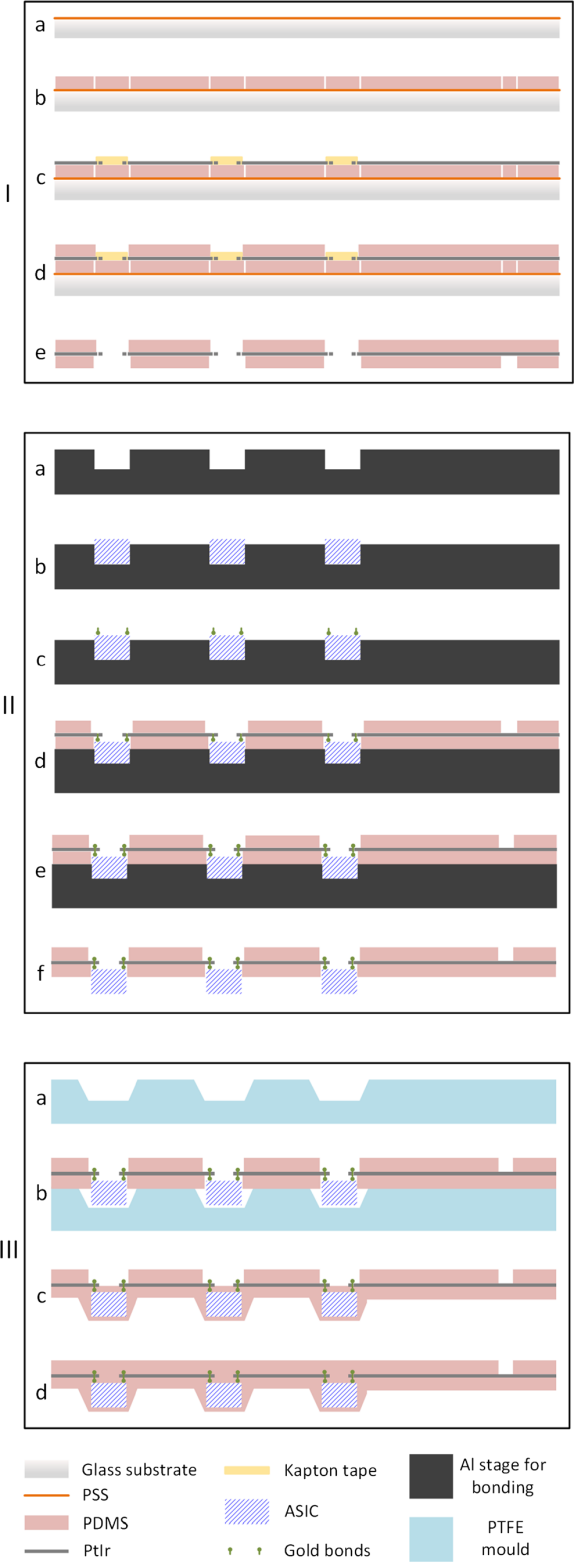
Schematic representation of the complete fabrication flow: **(I)** fabrication of the electrode interconnects, (**II)** ASICs microassembly, and **(III)** final encapsulation of the device

Figure 6 graphically illustrates the three processes of the complete fabrication flow, and their discrete steps, which are discussed below.

#### Fabrication of the electrode interconnects

In Fig. 6(Ia), a layer of poly4-styrenesulfonic acid solution (PSS) (Sigma-Aldrich, USA) (orange) is deposited on a glass slide (light grey). The glass slide serves as the mechanical carrier throughout this part of the process, while the PSS is a sacrificial layer, allowing eventual release of the device when needed, without damaging it. A layer of PDMS [the two parts of PDMS were mixed for 2 min at 2700 revolutions per minute (rpm) using a Speed Mixer (DAC 150 FVZ-K, Hauschild Engineering, Germany)] is first deposited [70 μm thick, spin coated, (WS-400BZ-6NPP/LITE, Laurell Technologies, USA)] in two phases; a low speed dispense step (15 s at 200 rpm) and a high speed step (45 s at 2000 rpm) during which the uniform layer is formed. The PDMS was cured in an oven at 50 °C for 1 h. On the resulting layer the openings for the electrodes, pads and holes for the chips are formed [Fig. 6(Ib)]. This is done by laser-cutting (Nd:YAG Class IV laser, Laservall S.p.A., Italy) the perimeter of these openings, rather than completely hatching (removing) the PDMS at this point. The fact that cured PDMS will be present during the formation of the second PDMS layer helps to prevent the uncured PDMS from flowing into the holes. The unwanted PDMS can be removed by hand after the assembly of the device has been completed to expose the electrodes and pads. The laser parameters used for the shaping of PDMS are listed in Table 2.

**Table 2:** Laser parameters

	PDMS	PtIr	
		1st passage	2nd passage
Power	49 %	40 %	40 %
Shot frequency	4400 Hz	4000 Hz	4000 Hz
Scan speed	60 mm/sec	2.1 mm/sec	7.5 mm/sec
Passes	380	1	1
Filling type	no filling	no filling	no filling
Filling angle	–	–	–
Filling spacing	–	–	–

In [Fig. 6(Ic)] the metal foil (dark grey) is next carefully laminated on the PDMS surface using a paddle roller. The laser (the parameters used for PtIr are also in Table 2) forms the electrodes, tracks and contact pads according to the specific design (Fig. 3). It is estimated that a minimum hole size and a minimum gap between tracks of about 20 μm can be achieved with this laser processing. The metal cutting is performed in two stages. First, the tracks close to the chip are shaped. The excess metal is removed by hand and Kapton tape (yellow) is attached on the already cut metal surface. This is necessary to hold the tracks in place during further handling. Next, the rest of the metal is shaped and any excess metal is again removed by hand.

The second (also 70 μm thick) layer of PDMS is then spun initially uniformly along the surface of the device, but post processed to keep the top surface of the Kapton tape free [Fig. 6(Id)]. The removal of the unwanted PDMS is done by hand before curing using a surgical blade. During this step, the narrow gaps between the metal tracks should be filled with PDMS to ensure complete electrical isolation from each other. Air could be present in the form of a bubble in the PDMS and this will be a potential weak point for the final implant. Thus, after mixing, the PDMS is inserted for a couple of minutes in a vacuum centrifuge (spun at 500 rpm, 80 g acceleration, at 60 mbar pressure) to remove trapped air.

After the second layer has been formed, the device is released from the substrate and the PDMS that was present where the ASICs are to be placed is removed [Fig. 6(Ie)]. At this stage, both sides of the metal tracks close to the ASICs are accessible for bonding.

#### Microassembly of the ASICs

The integration of the ASICs on the rest of the device [Fig. 6(II)] presents several challenges. The ASICs need to be positioned in specific places so that they are well aligned to the rest of the device, and they need to stay fixed there securely during bonding, but easily released afterwards to avoid damaging the (still unprotected) bonds.

**Fig. 7:**
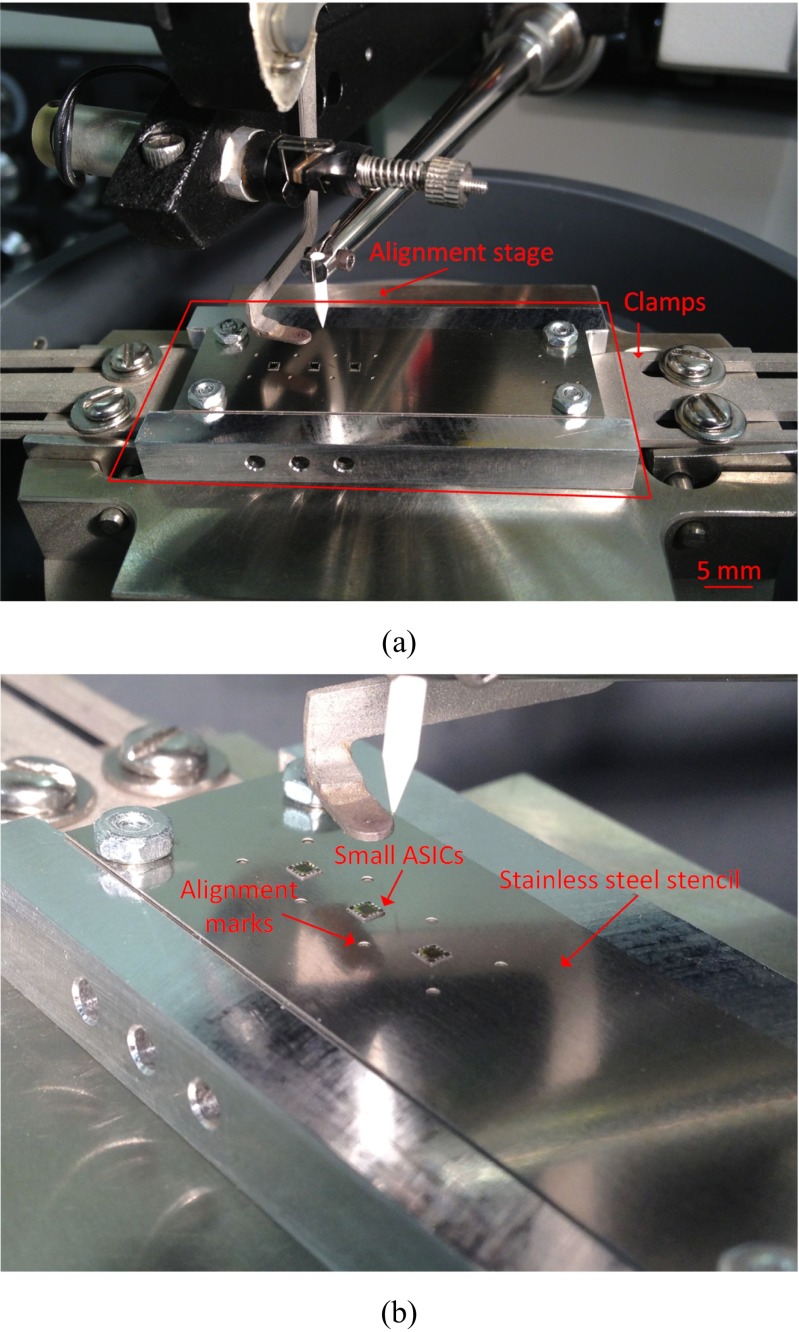
Alignment stage used during the integration process; (**a**) stage clamped on the wire bonder, and (**b**) detailed view of the stencil fixed on the base of the stage. The three ASICs and the extra alignment marks are visible

To align the parts, a dedicated stage has been designed [Fig. 6(IIa) black structure, and Fig. 7]. The structure comprises a base and a top stencil. The base is made from aluminum and can be clamped on the wire bonder where it can be heated up to the desired temperature. The stencil is made from stainless steel foil (80 μm thick) and contains holes for the ASICs allowing for a gap of approximately 5 μm around them. In addition, extra marks and threaded holes have been included, to ease the visual alignment of the different parts and ensure stable fixation, respectively.

During the bonding process, the ASICs are securely attached (hotmelt thermoplastic adhesive Techbond 7718 (Power Adhesives, UK) on the alignment stage [Fig. 6(IIb)] and gold studs are formed on the pads [Fig. 6(IIc)].

Next, the array is aligned on top of the ASICs so that the holes on the metal tracks are placed directly on top of the first gold stud [Fig. 6(IId)], and a second gold stud is formed through the hole [Fig. 6(IIe)], completing the electrical rivet bonding. Provided the ASICs are securely attached on the stage, we found that 93 % of the bonds were successful during the first attempt. The array is then released from the stencil [Fig. 6(IIf)]. At this point, the three ASICs are bonded on the flexible device but both their top and bottom surfaces are exposed.

#### Final encapsulation and external connections

To complete the fabrication process the ASICs need to be encapsulated in PDMS. The encapsulation ensures that the conductive parts are isolated and are not in contact with the tissue, and it also provides mechanical support to the bonds, resulting in a more reliable device.

##### Bottom encapsulation

**Fig. 8:**
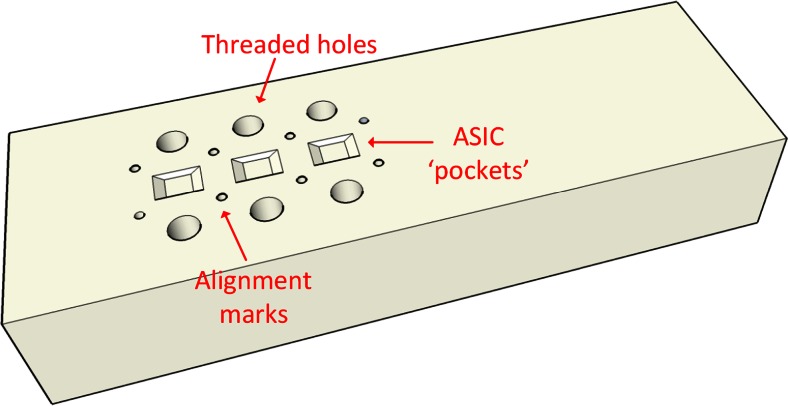
Encapsulation mould

To keep the thickness of the device within the desired limits and to ensure no air bubbles are formed during the encapsulation stage (this would compromise the lifetime of the implant) a custom-made mould was used in this part of the process [Fig. 6(IIIa) (blue), and Fig. 8]. This contains three pockets for the ASICs which are filled with uncured vacuum centrifuged PDMS before the active structure is placed on top (with the backside of the chips inside the pockets). It is made of PTFE, so that the PDMS can be easily removed from it after curing. Three metal strips are used to fix the device in place through the threaded holes. After curing, the ASICs are covered with an approximately 25 μm thick layer of PDMS and the array is very easily removed from the mould without pulling.

##### Top encapsulation

In the final step, the electrodes are covered with Kapton tape for protection, and some extra PDMS is deposited [so that the top layer of Fig. 6(IIId) is flat] using a fine nozzle to cover the top surface of the chips and then spun to get it uniformly distributed. It is heated under pressure to prevent bubbles from forming during the cure.

##### External connection

For the connection of the array to the stimulation control unit, stainless steel wires (Cooner AS632, USA) are soldered on the pads (right side of Fig. 3), and this area is covered with some extra PDMS, which can be completed during the top encapsulation step.

#### Prototypes

**Fig. 9:**
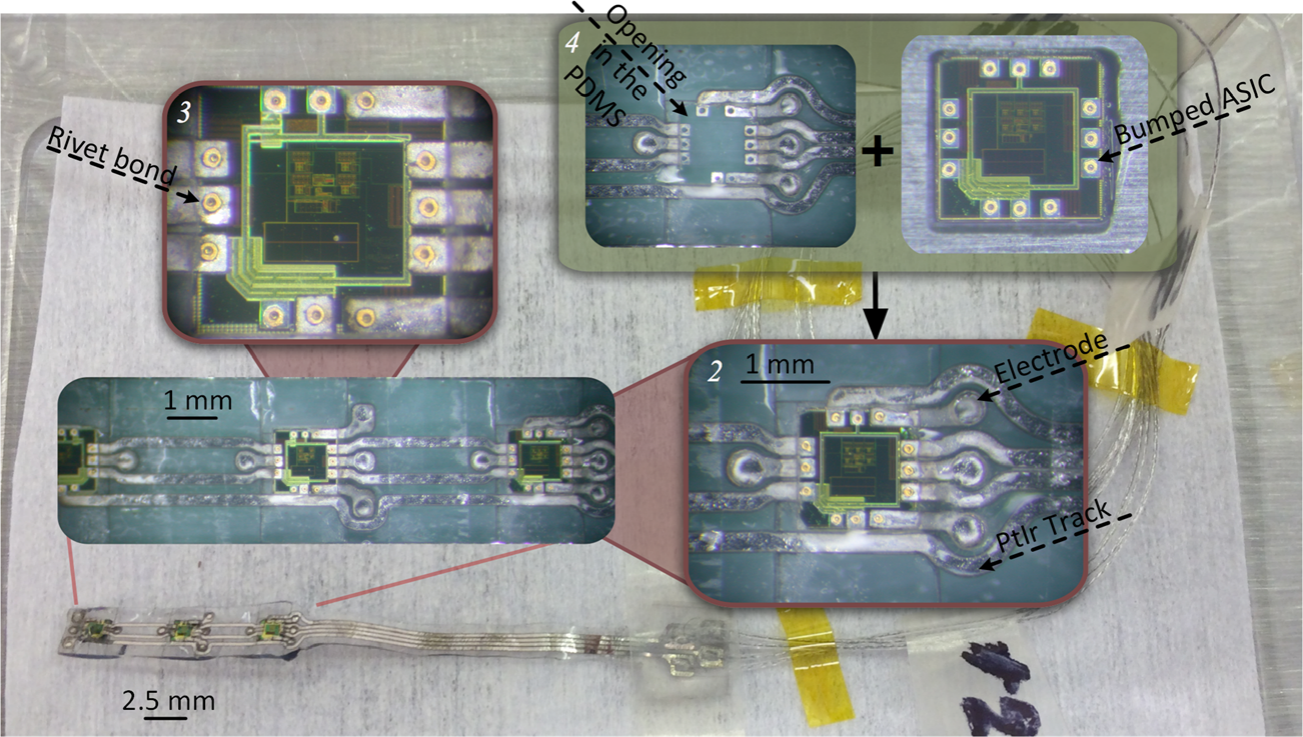
Complete fabricated prototype (background) with wires attached and detailed views of the electrodes, metal tracks and bonded ASICs [before final encapsulation as in Fig. 6 (IIf), insets **1**, **2** and **3**]; Inset **4** shows the electrode interconnects with both sides of the metal tracks accessible for bonding **(** ***left*** **)** and the bumped ASIC **(** ***right*** **)** before microassembly – as in Fig. 6 (Ie) and (IIc)

**Fig. 10:**
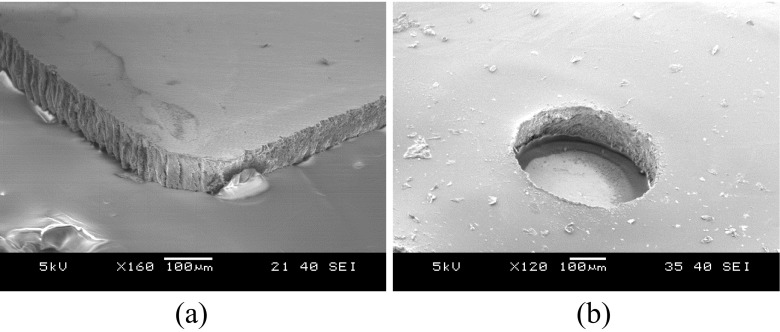
SEM images of the laser-cut ASIC corner (**a**) and the PDMS rim around the PtIr electrode (**b**)

Figure 9 shows details of the prototypes at different stages of the fabrication flow. Figure 10a shows a scanning electron microscope (SEM) image of a laser-cut chip corner and the smooth backside surface produced using the thinning method described in section 2.3.1 (for this image a ~60 μm thin chip was used). The laser-cutting of silicon ASICs allows the formation of arbitrary die shapes, as opposed to saw dicing, but results in rougher chip side walls (please refer to Fig. 17 in Kröninger and Mariani (2006) for a comparison of chip side walls separated by these two different methods). Alternatively, the dicing-by-thinning method with dry-etched trenches has been shown to produce even smoother edges [Fig. 5 in Schönfelder et al. (2007)], leading to increased chip strength, for applications where wafer level processing is possible. Figure 10b illustrates an SEM image of the laser-formed opening in the PDMS over a PtIr electrode. The PDMS laser cutting creates clear lines without any extra noticeable deformation.

### User control

**Fig. 11:**
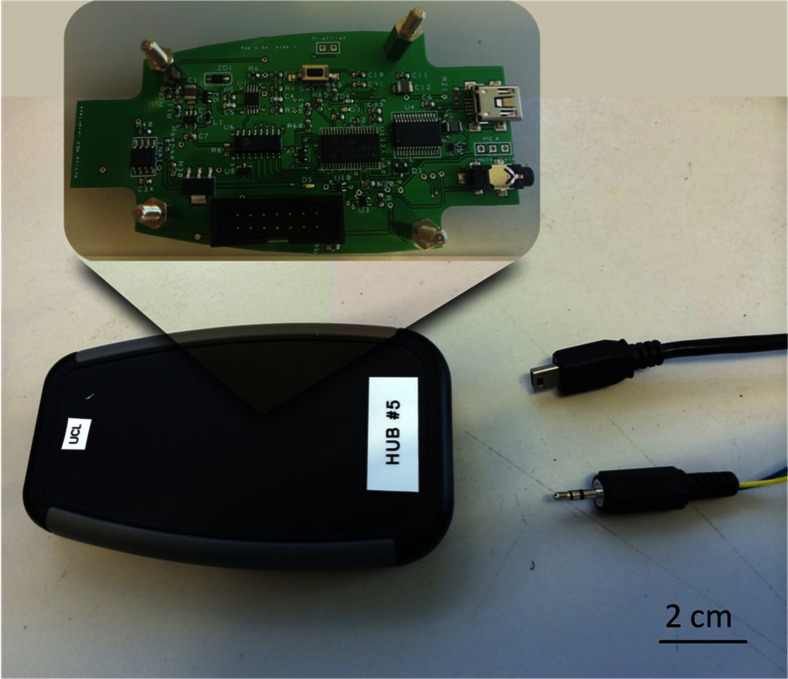
External control unit; a USB cable connects the unit to a PC from which it receives power and the stimulation parameters defined by the user on the GUI. A stereo audio plug holds the three connecting lines for the communication of the unit with the active array

The stainless steel wires soldered on the array pads connect to an external control unit which is used to store the externally defined parameters, and generate the stimulus current as well as control the timing of the stimulation. The unit comprises a microcontroller, a current source, a dc-dc converter and several other components on a PCB (Giagka et al. 2014c), and is powered through a USB connected to a PC (Fig. 11). A graphical user interface (GUI) serves as the tool through which the user can modify the stimulation protocols. The GUI gives the user the freedom to select up to five different stimulation patterns and independently define their individual parameters (pulse amplitude, pulse width, interphase delay) and electrode configurations. In addition, the user can define the stimulus repetition rate; all patterns will be then applied in a sequential manner, with the same period or a multiple of the smallest period. The external control unit and active electrode array system is capable of a swift change of parameters (in microseconds) and could be dynamically adjusted in real-time several times during a gait cycle, providing great versatility for testing.

### Mechanical tests

**Fig. 12:**
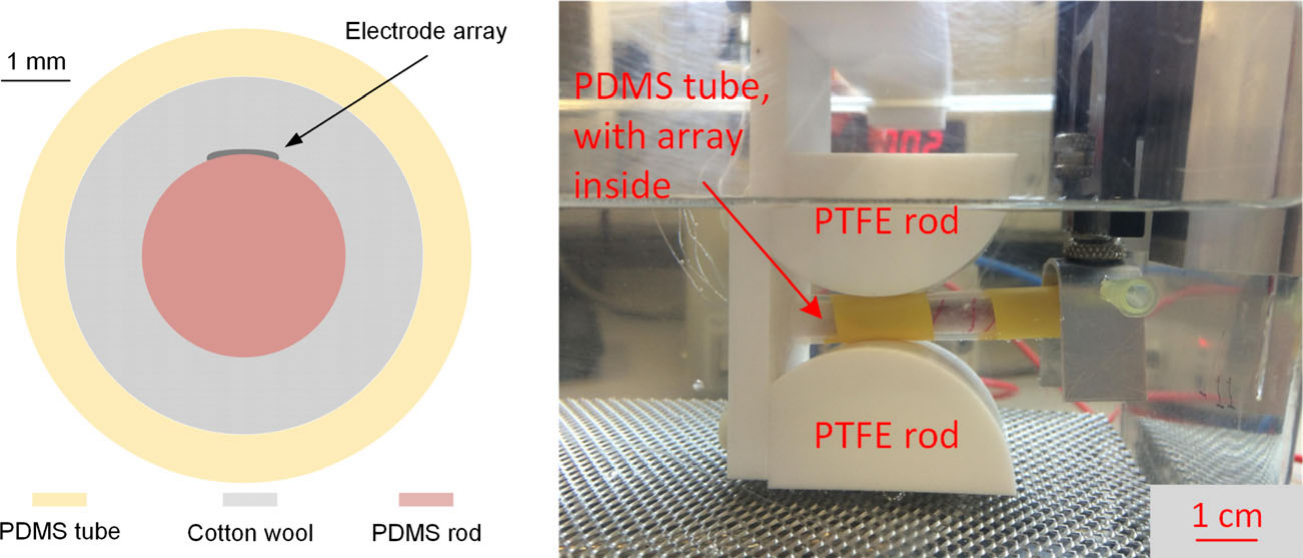
During the bending test the electrode array is placed inside a PDMS tube **(** ***left*** **in cross-section)**, which, in turn is trapped between two PTFE rods **(** ***right*** **)**. A motor is used to move the PDMS tube up and down, thus inducing controlled bending of the array around the rods

To test the mechanical properties of the prototype devices a custom made setup was developed that would mimic—as accurately as possible—the worst case chronically-repeated unidirectional deformation to which implant would be subjected. Observing how rats move in a cage, it was concluded that the most extreme deformation would come from either flexion or extension. This is supported by both *in vitro* and *in vivo* measurements of the maximum angular displacement of the lumbar spine in Cunningham et al. (2010). The setup induces controlled bending of the active array during electrical stimulation in normal saline at body temperature; two half PTFE rods (Fig. 12) are used to set the bending radius to 25 mm. The electrode array is first attached to a 3 mm diameter PDMS rod (mimicking the spinal cord), and is surrounded by cotton wool, which allows the saline to reach the electrodes. A cotton thread is wrapped around the array to fix it in place. The whole PDMS rod-array-cotton structure is placed inside a larger PDMS tube (of 6 mm inner diameter), which is trapped between the two PTFE rods. A motor is used to move the PDMS tube up and down (1 cycle per second) by 4 mm (total vertical movement), thus inducing controlled bending of the array around the rods. The whole structure is immersed in normal saline and current is delivered to all electrodes, in pairs, sequentially. The temperature of the saline solution is kept at approximately 36 °C to emulate the body temperature.

### Electrical characterization

To characterize the electrical behavior and properties of the new arrays, access to the individual electrode sites was needed. This is not possible once the active array has been fabricated, since the ASICs controlling the electrodes are now fully encapsulated. Nevertheless, it was possible to test a passive version of the array fabricated with the same material (for this purpose a PtIr passive array with the same electrode size and arrangement was produced). This is interfaced via stainless steel wires to a PCB where the ASICs are mounted, and through which the desired sites become accessible. Of course, small deviations from the measurements are expected for the active implant, but they should not be significant.

## Results

### Mechanical tests

The tested prototype had nine functional electrodes out of the 13. This was due to a failed connection during fabrication on the V_SS_ line of one of the ASICs. We subjected the device to continuous bending for 14 days, during which we applied electrical stimulation (500 μA, 200 μs) for a total of 21 h. No failures were observed after the end of the test (over 1.2 M bending cycles), either at any of the – initially good – bonds, or at any of the tracks. The good continuity was also verified electrically. In addition, examination under the microscope revealed that the metal of the electrode contacts and tracks had not degraded in any visible way.

### Electrical characterization

**Fig. 13:**
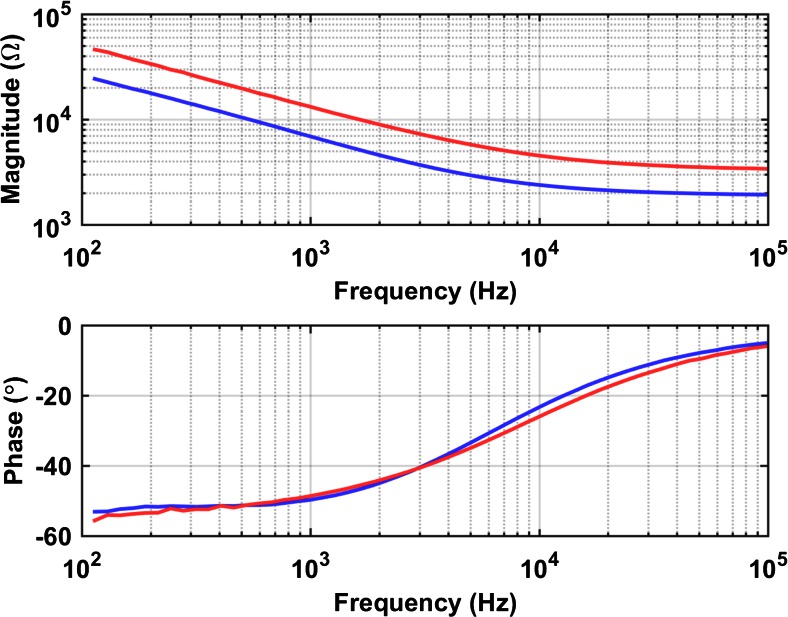
Typical measured impedance spectra (magnitude and phase) for monopolar **(** ***red*** **)** and bipolar **(** ***blue*** **)** electrode configurations

Figure 13 shows typical (monopolar and bipolar) impedance spectroscopy traces of electrodes in unbuffered saline. The exposed 1 cm tip of a stainless steel wire was used as the return electrode for the monopolar measurements. Measured standard deviations of electrode impedance from all PtIr electrodes at 1 kHz is 0.4 and 0.6 kΩ for the monopolar and the bipolar case, respectively.

**Fig. 14:**
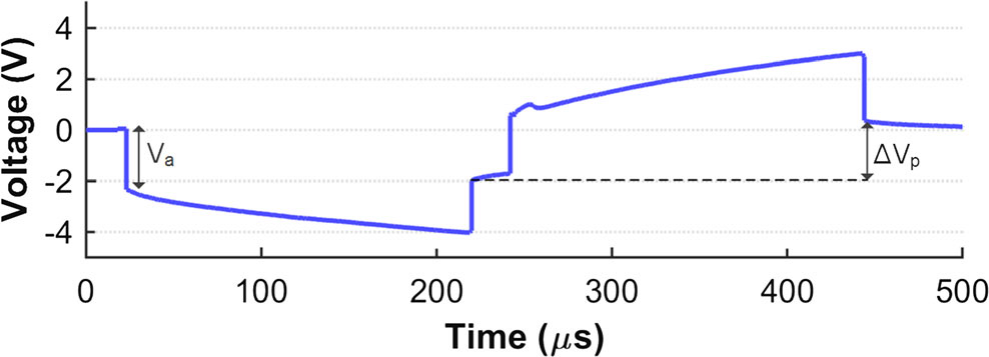
Measured voltage waveform between the cathode and the anode electrodes for a 1 mA stimulation with maximum pulse widths of 200 μs. For this recording one electrode on the array was selected in a monopolar configuration

Figure 14 shows the typical shape of the recorded voltage waveform during 1 mA biphasic pulsatile stimulation for electrodes when used in monopolar configuration. The 1 mA test current exceeds the maximum current typically used in rat ESCS (600 μA, typical stimulating parameters for this application are discussed in Capogrosso et al. 2013); it was, nonetheless, selected, to investigate the limits of the active implant (the embedded ASICs and electrodes were designed to accurately and safely deliver currents up to 1 mA).

When a PtIr electrode is immersed in an electrolyte containing chloride, the maximum voltage swing before oxygen and hydrogen evolution is about 2.15 V (Vanhoestenberghe 2007). However, this voltage swing is usually extracted from cyclic voltammetry studies at low current densities, and, Donaldson and Donaldson (1986) report that, in practice, if the safety of the stimulation is based on bubble formation (which is the indication of electrolysis), this limit can be extended in both directions, implying that this voltage swing is actually larger. Similarly to the analysis in Giagka et al. (2014c), the potential difference ΔV_p_ of Fig. 14 can be related to this voltage swing; this suggests that currents of 1 mA and below are within the limit for safe stimulation with this system. The shape of the waveform suggests that the nature of these electrodes is strongly capacitive, however not purely, and this is also supported by the impedance phase graph, which at lower frequencies is closer to −55° (a pure capacitor would be −90°). Similar tests conducted using gold electrodes of similar size revealed a larger capacitive element (Giagka et al. 2014c). Finally, the impedance value at high frequencies indicates an expected resistive contribution of the electrolyte about 2 kΩ, and this is also observed in Fig. 14 (value V_a_ is close to 2 V for 1 mA).

## Discussion

These active arrays still need an external stimulation control unit in this version. However, the developed technology, presented here, serves as the first step towards a future ‘mobile’ version in which the existing control unit will be miniaturized subcutaneous, and battery-powered.

The novel implant layout with ASICs inside the rat’s spinal canal could also be useful in cases when recording of neural signals is needed (which would require the addition of the sense circuits), for example, for a closed-loop system as it reduces noise introduced by long tracks on the recorded signal.

The limitation of the current implant layout is the fact that all ASICs are connected together serially. As a result if a failure occurs in one of the input lines of the first-in-the-series ASIC, the failure will affect all the following ASICs. Due to the limited area available for this application, this type of connection was selected as a trade-off for a smaller implant width. If that was not of such concern one could modify the track design to make sure that all chips’ input lines are connected in parallel, thus isolating the failures only to the ASIC in which they occurred.

Regarding the fabrication of the active arrays, one of the most challenging stages was the temporary fixing of the small dice on the wire bonder during the assembly process. The bonding of the ASICs requires that the dice are very well attached on the substrate during the procedure [Fig. 6(IIb)]. This, although straightforward for larger devices, is difficult for small dice. Clamping is not an option and the retention level that one could achieve using vacuum proved to be insufficient for this application due to the limited surface area. An adhesive material was sought that would satisfy the following requirements: i) provide a strong bond, stable throughout processing at the elevated temperatures required for bonding (up to 150 °C), ii) would not cause damage to the chip, and iii) would be easily – and completely – removable, leaving a clean surface, ready to be encapsulated. The selected hotmelt thermoplastic adhesive, is inexpensive, readily available, readily soluble to isopropanol and acetone and provides the necessary retention strength if the bonding temperature is lowered to 120 °C (rather than 150 °C). Although, according to the product datasheet the softening point is specified to be 160 °C, in reality, the softening process has already started at about 130 °C. The effect of this lower temperature on the bonding strength is unknown, and more pull tests (Giagka et al. 2014b) are required to gain a better understanding of this issue. Alternatively, specially designed adhesives (fabricated and produced on order) might allow bonding at higher temperatures.

An initial *in vitro* electrical and mechanical evaluation of the implant’s performance has been presented. The electrical evaluation indicated that safe stimulation is possible; the low power operation of the ASICs ensures that the dissipated heat will not cause tissue damage [by respecting the limit for implantable devices of a maximum of 400 μW/mm^2^ chronic heat flux (Davies et al. 1994) – the 114 μW is dissipated through the 3 ASICs, that is through at least an area of 3 × 4 mm^2^ = 12 mm^2^, which is approximately 9.5 μW/mm^2^]. The results of the chronic bending tests revealed no bond or track failures on the tested prototype. These results are promising regarding the chronic performance of the devices. Nevertheless, the movements of the rat during a day are complex and the spinal cord can be subjected to a combination of bending, some stretching, and possibly torsion. A test device that accurately mimics these movements might give a more reliable estimate regarding the real performance of an array implanted inside the spinal canal. In addition, the integrity and lifetime of the insulation layer when the implant is used for stimulation would need to be tested. However, it has been shown that PDMS encapsulation of ASICs can have a lifetime of several years (e.g., Fig. 5 in Vanhoestenberghe and Donaldson 2013). Chronic *in vivo* testing of the devices will be the topic of future work.

During chronic implantation the tissue response will increase the load/electrode impedance, and as a result higher voltages will be required to deliver the same/requested charge. In current controlled stimulation systems as the one presented here, if the voltage compliance of the system (in this case approximately 25 V) is high enough to accommodate for these impedance changes, the correct charge will always be delivered to the load.

The voltage waveform in Fig. 14 suggests that this system can safely deliver the requested maximum charge to the tissue. However, if needed, processing of the PtIr surface can be employed (porous surface and/or coatings such as activated iridium oxide) to improve the charge transfer capabilities of the electrodes.

## Conclusion

A novel design and fabrication process for flexible active stimulating epidural electrode arrays for rats has been proposed. The 12-electrode PDMS-based array includes three small, thin ASICs that are suitable for implantation inside the rat’s spinal canal. Thanks to the ASICs, using only three communication wires independent access can be provided to all 12 PtIr electrodes in any possible configuration. Chronic bending tests with a custom setup on one prototype revealed no bond or track failure. The electrical evaluation of the devices suggests safe stimulation. The active arrays can be electronically programmed via a GUI that communicates with an external control unit connected to the implant. Stimulation parameter adjustment is rapid, and can be done several times during a gait cycle. This feature, together with interleaved stimulation supported by the hardware, ensure full testing protocol versatility.
